# Supplementary material for: Effects of Altering Magnesium Metal Surfaces on Degradation In Vitro and In Vivo during Peripheral Nerve Regeneration
Source: Materials (Basel). 2023 Jan 30;16(3):1195. doi: 10.3390/ma16031195 (PMC9920421; doi:10.3390/ma16031195)
Supplement: Supplementary file 1 [file materials-16-01195-s001.zip › materials-2156967-supplementary.pdf]

# Effects of Altering Magnesium Metal Surfaces on Degradation In Vitro and In Vivo During Peripheral Nerve Regeneration

**Supplementary Materials**

**R. Tatu, L. White, Y. Yun, T. Hopkins, X. An, A. Ashraf, K. Little, M. Hershcovitch, D. Hom, and S. Pixley**

.

# Supplementary Materials

## Supplementary Figure S1

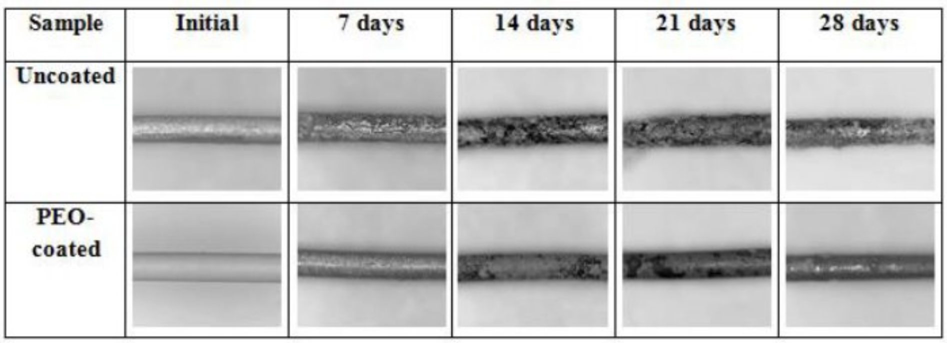


**Figure S1.** Optical images of degradation in vitro. PoI (uncoated) and PEO (PEO-coated) Mg filaments were imaged prior to and weekly through 28-day immersion in HBSS. Microscopic examination under similar exposure conditions showed that several optically distinct corrosion products were present on the magnesium sample surface at most of the time points. After immersion for 7 days, the unpolished sample begins to show significant cracking and corrosion pits., while for the PEO-coated sample, it is difficult to see any sign of major corrosion damage. Thus, the PEO coating was able to provide initial corrosion protection. As time progressed, severe localized corrosion continued to develop for the unpolished sample as the corrosion pits became increasingly deeper and the diameter of the unpolished sample appears to get reduced over time. A thin, white surface layer is deposited on the surface of both the unpolished and PEO coated samples during the 28-day period. With copyright permission from [1].

**Supplementary Figure S2**

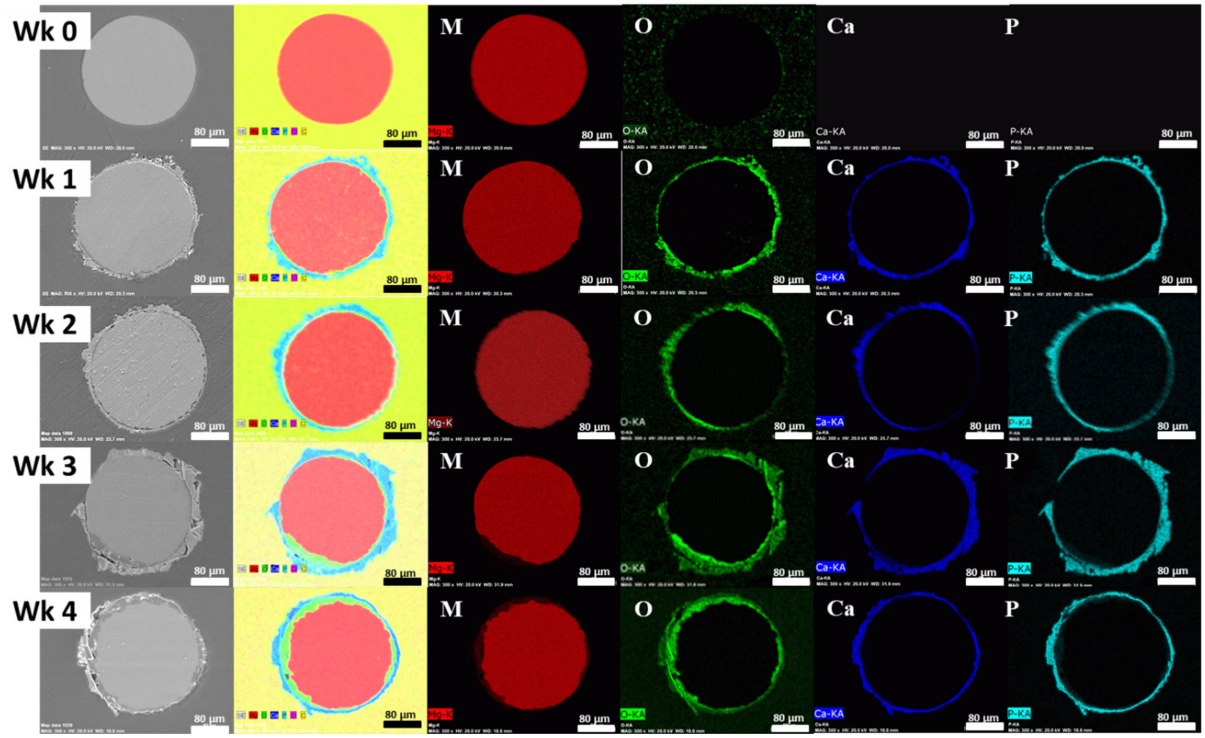


**Figure S2.** SEM and EDX Images of Pol filament degradation in vitro. SEM images (column 1) and EDX mapping (columns 2-6) were performed before and every week after immersion in HBSS. Column 2 shows merged images of all EDX maps. Columns 3 – 6 show EDX maps of Mg, oxygen (O), calcium (Ca) and phosphorous (P). Corrosion and surface deposits increased over time. Note, very little surface deposits at week 0, before immersion. Bars = 80 µm. With copyright permission from [1].

**Supplementary Figure S3**

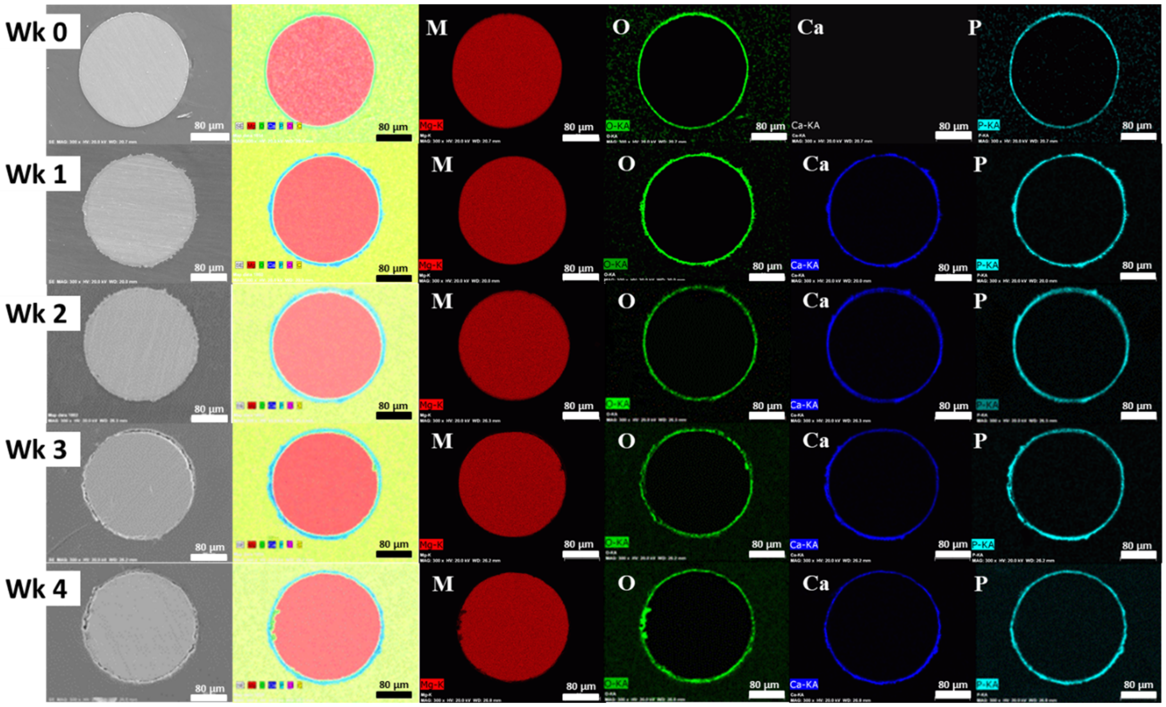


**Figure S3**. SEM and EDX Images of PEO filament degradation in vitro. SEM images (column 1) and EDX mapping (columns 2-6) were performed before and every week after immersion in HBSS. Column 2 shows merged images of all EDX maps. Columns 3 – 6 show EDX maps of Mg, oxygen (O), calcium (Ca) and phosphorous (P). Corrosion and surface deposits increased over time. Note, PEO anodized layer is detectable at week 0, before immersion. Bars = 80 µm. With copyright permission from [1].

## Supplementary Table S1

**Table S1.** Measurement of EDX elemental spectra in vitro. This table provides the EDX measurements of the chemical composition of the surfaces of uncoated (Pol) and coated (PEO) Mg filaments before and after 28-day immersion in HBSS. With copyright permission from [1].

| Sample | Mg | O | Ca | P | Cl | C | Ca/P ratio | Ca/P ratio SD |
|---|---|---|---|---|---|---|---|---|
| Uncoated (Initial) | 93.87 | 5.43 | 0 | 0 | 0 | 0.63 | 0 | 0 |
| PEO (initial) | 23.72 | 39.16 | 0 | 1.24 | 0 | 34.7 | 0 | 0 |
| Uncoated (28 days) | 5.94 | 49.13 | 13.18 | 9.36 | 0 | 23.09 | 1.53 | ± 0.18 |
| PEO (28 days) | 2.526 | 46.77 | 17.45 | 9.76 | 0 | 22.62 | 1.76 | ± 0.17 |

**Supplementary Figure S4**

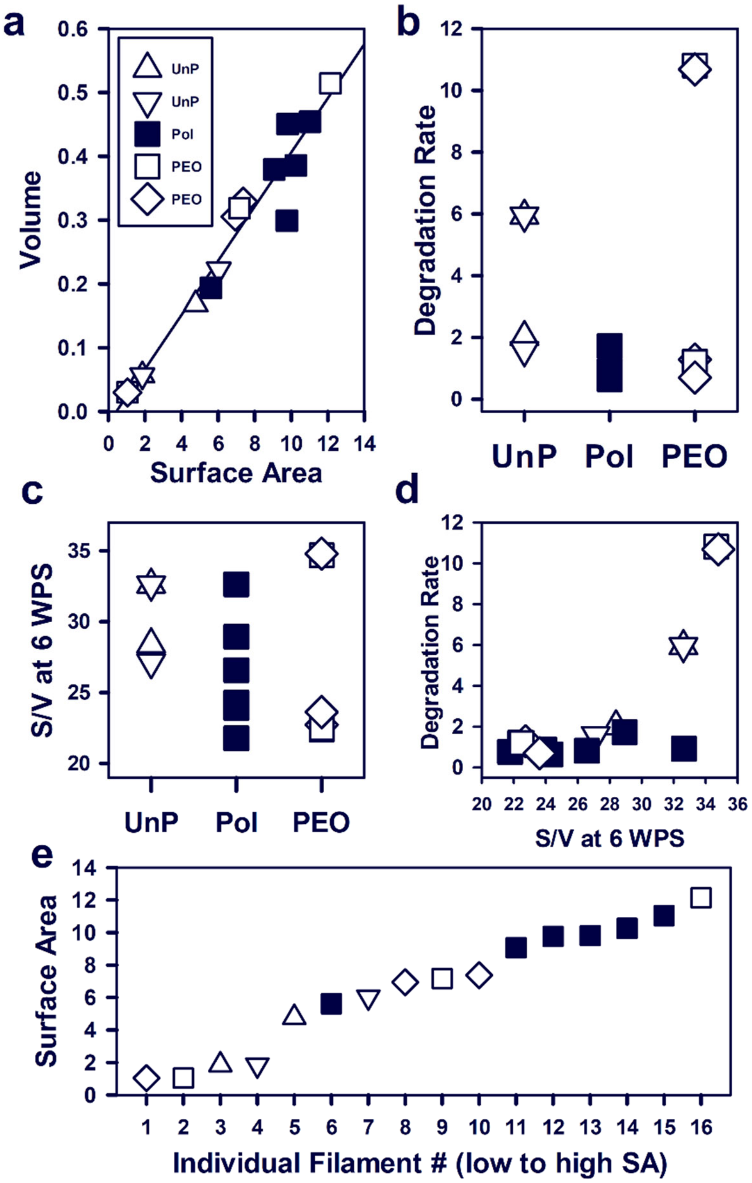


**Figure S4.** Measurements of filament degradation in vivo. Symbols for UnP and PEO were given alternating symbols to allow visualization of overlapping values (legend in a). Filaments were imaged via micro-CT prior to implantation and in live animals at 6 WPS and quantified to provide graphs of (a) surface area vs volume at 6 WPS; (b) degradation rates (DR) per group (including animal in Fig. 6e) (Kruskal Wallis ANOVA on ranks, p = 0.05, Dunn's with UnP as Control: Pol vs Unp, p = 0.037; PEO vs UnP, p = 0.742). (c) Surface area/ Volume (S/V) per group (ANOVA p = 0.475). (d) Degradation rates were roughly related to S/V values. (e) Surface area per individual filaments after ranking allows visualization of overlapping values.

**Supplementary Figure S5**

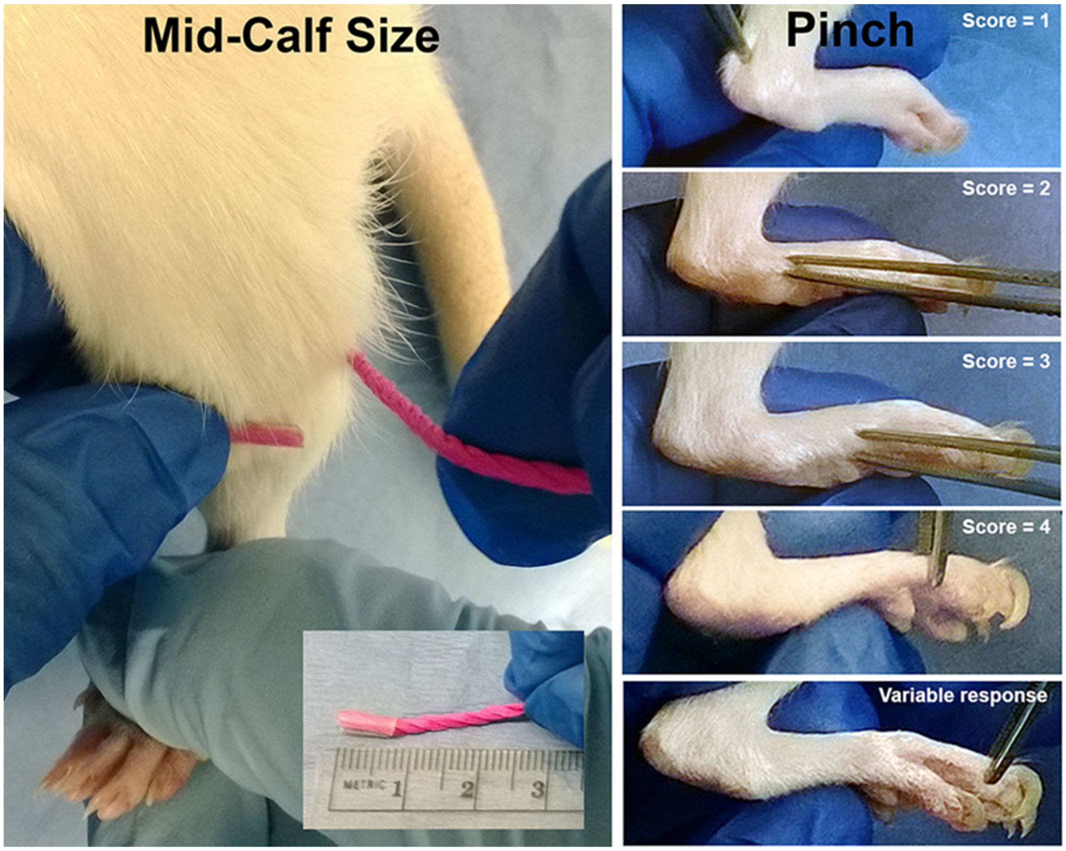


**Figure S5.** Live animal analysis of calf circumference and foot pinch. Left (a) Calf circumference was measured with a thread kept at the belly of the calf muscle. Right (b) Pinch test scoring. Responses were retraction or vocalization. Scoring of toes 1-4 was highly variable.

## Supplementary Table S2

**Table S2.** Scoring for reflex toe movement (toe spread) and foot pinch tests. No animal exhibited a score of four for toe movement, while isograft, but not conduit, animals achieved levels of 4 for foot pinch.

| Scale | Reflex Toe Movement | Foot Pinch |
|---|---|---|
| 0 | No movement: held tightly against toe #4 | No response anywhere on ankle or lateral foot |
| 1 | Slight movement | Response for skin at back of ankle |
| 2 | Toe curling or lateral movement | Response at ankle plus skin on lateral foot, rear side |
| 3 | Distinct Lateral movement + some uncurling (extension) | Response at spots above plus skin on lateral side, front, beside ball of foot |
| 4 | Normal: lateral movement and extension | Response at spots above and toe #5, pinching bone between joints |

## Supplementary Table S3

**Table S3.** Tissue continuity (TC) and neurofilament (NF) scales. For TC, the scale was judged by viewing axial sequences of images from iodine micro-CT (>1,000 images per rat tissue) in 3D with ImageJ. Neurofilament (NF) scale: NF-positive fibers (axons) in immunostained cross sectional images were compared for relative amounts and scaled. For each scale, analysis was done by two or three observers blinded to experimental conditions.

| Scale | Tissue continuity (TC) | Neurofilament + Fibers (NF) |
|---|---|---|
| 0 | Tissue clearly did not get through | No NF+ fibers |
| 0.5 | Very difficult to find, but tissue could get through. Conduit very disrupted | |
| 1 | Difficult to find, but tissue could get through. Conduit shattering likely | Very few, scattered, around 10 |
| 1.5 | Tissue goes through, >90% constriction | |
| 2 | Tissue goes through, 75 - 90% constriction. | Small Patch of fibers, between 10 and uncountable (~50) |
| 2.5 | Tissue goes through, 50 - 75% constriction. | |
| 3 | Tissue goes though, around 50% constriction | Larger patch fibers (can't count) or > 1 smaller patches |
| 3.5 | Tissue goes through, around 25 - 50% constriction. | |
| 4 | Tissue goes through. with one major conduit break (< 25%) + other breaks. | Big patch, might be similar to one small, normal fascicle |
| 4.5 | Tissue clearly goes through. Conduit has one break with slight constriction | |
| 5 | Theoretical upper limit. No breaks in conduit. Tissue with no constriction. | Even bigger patch, size up to one medium size fascicle |

### References:


1. White, L.G. Investigation of Coatings with Controlled Degradation for Biodegradable Magnesium Implants. ProQuest Ph.D. Thesis, 2014, North Carolina Agricultural & Technical State University, Greensboro, NC USA.
